# Quality of Spirometry Maneuvers in Interstitial Lung Disease Patients in a Tertiary Facility

**DOI:** 10.3390/jcm15218253

**Published:** 2026-10-23

**Authors:** Denis Vinnikov, Aida Zhadil, Saule Kassenova, Rustem Tuleutayev

**Affiliations:** 1 Environmental Health Laboratory, Al-Farabi Kazakh National University, Almaty 050040, Kazakhstan; denisvinnikov@mail.ru; 2 Occupational Health Risks Laboratory, Peoples’ Friendship University of Russia (RUDN University), 117098 Moscow, Russia; 3 Department of Internal Medicine No. 2, Research Institute of Cardiology and Internal Diseases, Almaty 050000, Kazakhstan; 4 Department of Internal Medicine, Asfendiyarov Kazakh National Medical University, Almaty 050012, Kazakhstan; 5 Department of Internal Medicine, Research Institute of Cardiology and Internal Diseases, Almaty 050000, Kazakhstan; skassenova07@gmail.com (S.K.); rustemtuleutayev@gmail.com (R.T.)

**Keywords:** spirometry, pulmonary function testing, lung function testing, quality criteria

## Abstract

**Background/Objectives:** The aim of this study was to analyze spirometry quality and compliance with the current protocol in interstitial lung disease (ILD) patients at a tertiary-level facility in Almaty, Kazakhstan. **Methods:** We consecutively enrolled 125 patients, aged 60 (interquartile range (IQR) 52; 67) years, including 40 (32%) patients with idiopathic pulmonary fibrosis (ILD-IPF). Relaxed and forced maneuvers were completed, and we hereinafter analyzed vital capacity (VC), forced VC (FVC) and forced expiratory flow in one second (FEV1) both as z-scores and % predicted values in addition to number of attempts, ΔVC, ΔFVC, ΔFEV1, expiratory plateau volume and back extrapolation volume (BEV) in both maneuvers. Multiple regression was used to test predictors of these. **Results:** In this predominantly female cohort (73%), CAT (median 12; IQR 9; 18) and mMRC (median 2; IQR 1; 3) scores reflected high symptom burden with most suggestive of a restrictive pattern (62% of all, median FVC in the entire group −2.17; IQR −3.28; −1.4 z-scores or 68%; IQR 52.5; 80.5). The median VC and FVC maneuver attempts was 3. ΔVC was 0.05 (IQR 0.03; 0.09) L; ΔFVC was 0.07 (IQR 0.03; 0.12) L; and ΔFEV1 was 0.05 (IQR 0.02; 0.09) L, whereas expiratory plateau volume was 0.04 (IQR 0.02; 0.06) L. Ninety-six percent of tests were graded A or B based on ΔFVC and 98% based on ΔFEV1. **Conclusions:** This study showed that despite dyspnea and cough in ILD patients with severe symptom burden, a high quality of spirometric maneuvers can be obtained in the majority of patients.

## 1. Introduction

Interstitial lung disease (ILD) is a chronic, usually progressive and debilitating lung disease with poor prognosis [1,2,3]. This disease can develop both as an inflammatory process with a pre-existing connective tissue diagnosis, and without obvious inflammation, when fibrosis already manifests with symptoms. In most cases of an idiopathic process, the disease is progressing, but the rate of such deterioration is unpredictable [4]. In addition, the rate of progression of ILD, associated with connective tissue and autoimmune diseases, also depends on the primary diagnosis and treatment [5,6], but, overall, the disease rarely remains stable. 

Spirometry is a useful, non-invasive tool for monitoring the rate of disease progression and treatment effect, when forced vital capacity (FVC) is commonly used to compute the rate of annual decline. Unlike healthy subjects, patients with ILD experience accelerated FVC decline, conventionally classified as stable (less than 5% decline per year), marginal (≥5% and less than 10%), and significant decline (≥10%) [7]. Because spirometry testing relies solely on patients’ efforts, quality control is crucial in sustaining validity, repeatability and acceptability [8]. However, successful spirometry test completion for ILD patients may be even more challenging compared to healthy subjects given that these patients often suffer from dyspnea, cough and fatigue, sometimes even at rest [9,10,11,12]. Spirometry testing protocol [8] implies excellent compliance, but this may be hard to achieve when ILD patients can feel it is hard to make even a full inhalation, as required. In the general population [13], the required spirometry quality criteria can be problematic to attain, whereas ILD patients can feel it is hard to complete even a relaxed maneuver because of reduced lung volumes. 

In recent years, home lung function monitoring has become popular, and a wide array of publications report good to excellent test reproducibility at home compared to the office [14,15,16]. These studies also conclude that the quality of such spirometry is acceptable [16]. However, studies analyzing spirometry quality in this specific group of patients even in the best conditions of in hospital and outpatient centers are insufficient [4,7]. Little is known about whether ILD patients can achieve excellent quality given the usual dyspnea, cough and early fatigue they already experience at the time of testing. Therefore, we planned this study to analyze spirometry quality and compliance with the current protocol in ILD patients at the tertiary-level facility where these patients are hospitalized annually to monitor disease progression.

## 2. Materials and Methods

### 2.1. Study Venue and Patients’ Recruitment 

The study was approved by the local Institutional Review Board of the Research Institute of Cardiology and Internal Diseases (approval 2/2024 dated 27 November 2024), and all participants signed a written informed consent to participate following a detailed introduction. All included patients were hospitalized either for diagnosis verification or for treatment effectiveness monitoring and control, which is required at least once a year as per the local regulations. These in-patient subjects were enrolled in the study on a consecutive basis starting November 2024 and ending August 2026 with the diagnosis of ILD. The department had a capacity of 34 beds, including 8 for pulmonary disease patients, most of which were usually occupied by patients with chronic obstructive pulmonary disease (COPD) exacerbation or severe or uncontrolled asthma. The hospitalized patients group included both first hospitalization and the diagnosis was confirmed for the first time, as well as those with a history of the disease. 

### 2.2. Questionnaire

The questionnaire for this study was tested and used in our preceding studies, including nationwide population-based reports [13,17,18]. In the current study, the tool consisted of 49 items and included four major sections. These were a demographic part (date of birth, sex, highest attained education and family income); an environmental and occupational exposures part (lifetime occupational history, a few questions on smoking status, classifying into never, former and current cigarette smoking, followed by electronic cigarette and waterpipe use, exposure to secondhand (SHS) smoke, use of organic fuel for heating and cooking at home, and, finally, regular exercise 3 times a week or more); and a clinical part and specific quality of life part. In the clinical section, we included a COPD Assessment Test (CAT), modified Medical Research Council (mMRC) tool and a few questions on the history of the disease. The CAT consisted of eight items and scored from 0 to 40, and the mMRC tool was graded from 0 to 4. Both tools reflected the symptom burden. 

### 2.3. ILD Diagnosis

We used the local protocol for ILD patient management in Kazakhstan. Inclusion criteria included ILD confirmed earlier; no age limits; both sexes; and consent to participate. A common criterion for all hospitalized patients with the diagnosis was a combination of typical radiological attributes of ILD. However, the group included both patients with an idiopathic process and no other organ involvement, but pulmonary fibrosis, referred to as ILD-IPF hereinafter, and patients in whom a pulmonary interstitial process accompanied one of the verified systemic inflammatory diseases (abbreviated as ILD-SD). All patients underwent a high-resolution computer tomography (CT) scan, cardiac ultrasonography, pulse oximetry at admission, and a set of specific systemic inflammatory blood markers, including rheumatoid factor, anti-streptolysin-O, ANA, ANCA, ferritin and lactate dehydrogenase. Patients were classified into ILD-IPF with only CT signs of lung fibrosis and no other signs of a systemic inflammatory process. Whenever systemic markers were positive, we classified them as ILD-SD. 

### 2.4. Spirometry

In all patients, spirometry was completed within the first two days of hospitalization, if no contraindications were identified. No bronchodilation test was considered in this study. All patients rested for at least 20 min prior to the test, and test performance [8] along with test interpretation [19] followed European Respiratory Society (ERS)/Americal Thoracic Society (ATS) guidelines, accordingly. Patients were instructed in both Russian and Kazakh, depending on the preference, and all tests were completed in a seated position. These international guidelines for test performance were chosen because no local spirometry guidelines in Kazakhstan existed. 

We measured VC in a slow relaxed maneuver and FVC in a forced maneuver. Additionally, FVC fractions were also calculated, including forced expiratory volume in one second (FEV1), and their ratio. Both for slow and forced maneuvers, patients were required to complete at least three attempts, with at least three acceptable and repeatable maneuvers. Quality control criteria, including the number of attempts, number of acceptable maneuvers, ΔVC or ΔFVC and ΔFEV1, back extrapolation time and expiratory plateau volume (25 mL or less in the last 1 s of expiration), were monitored on the screen. In forced maneuvers, we aimed to achieve A or B scores in either or both for ΔFVC and ΔFEV1. We reported the actual volumes in addition to their z-scores calculated as the number of standard deviations from the predicted value, as well as the % predicted values. Percent predicted and z-scores were reported in this presentation concurrently. GLI-2012 was used as a set of predicted values. 

All spirometry tests were accomplished with an MAS-2PC (МАС-2ПК) device (Belintelmed LLC, Minsk, Belarus) running with pre-installed IntelSpiro software (version 4.2.0.9) on a notebook. We considered normal spirometry in patients with FEV1/FVC above the lower limit of normal (LLN, defined as −1.645 z-scores) and FVC above the LLN. Suggestion of a restrictive defect was defined as FVC below the LLN with normal FEV1/FVC. Finally, we also concluded an obstructive defect in some patients, when FEV1/FVC was below the LLN with normal FVC. 

### 2.5. Statistical Analysis

The primary outcome in this study was the prevalence of VC and FVC maneuvers of high quality, graded as A or B for ΔVC and ΔFVC (or ΔFEV1). In addition, we also assessed test quality via the number of attempts, ΔVC, ΔFVC, ΔFEV1 and Δ(VC-FVC) medians, as well as the back extrapolation volume (BEV) and expiratory plateau volume. All studied variables were first tested for normality, and most were found to be non-normally distributed. We, therefore, only used and reported non-parametric statistics in this study. We reported medians for all continuous variables with their corresponding interquartile ranges (IQRs). In the univariate comparisons, two groups were compared using χ^2^-test from the contingency tables for binary variables and the Mann–Whitney U-test for continuous variables. Such two-group comparisons were in place when ILD-IPF patients were compared to ILD-SD patients. When spirometry quality was analyzed, we tested three groups, including a normal pattern, suggestive of a restrictive pattern and obstructive defect. We then used the non-parametric Kruskall–Wallis test or χ^2^-test, depending on the variable of interest. 

We also used multiple regression models to test the association of selected predictors with the outcomes of interest, including the number of attempts, ΔVC, ΔFVC and ΔFEV1, BEV and expiratory plateau volume. In these models, a set of categorical (sex, education level, stratified into below college or university and above, cigarette smoking, and ILD-IPF vs. ILD-SD) and continuous variables (CAT score, mMRC score, age, body mass index (BMI), SpO_2_, respiratory rate and heart rate (HR) at admission) were tested first in the univariate regression. We reported beta-coefficients (β) for the variable of interest with the corresponding 95% confidence interval (CI). We considered *p*-values below 0.05 significant, and all models were completed in NCSS 2025 (Kaysville, UT, USA).

## 3. Results

Overall, there were 125 patients, aged 60 (IQR 52; 67) years, most of which had ILD associated with systemic disease. ILD-SD diagnoses included systemic scleroderma (N = 48, 56%), systemic scleroderma with rheumatoid arthritis (N = 10, 12%), systemic scleroderma with lupus erythematosus (N = 2, 2%), rheumatoid arthritis (N = 9, 10%), Sjogren’s disease (N = 9, 10%), dermatopolymiositis (N = 4, 5%) and mixed connective tissue disease (N = 2, 2%). This was a predominantly female cohort (Table 1) and its socioeconomic profile was generally representative of a typical Kazakhstan population, with more than 50% having education above high school. In general, current cigarette smoking prevalence was low, and more than 70% never smoked cigarettes. Current smokers reported smoking a median of 17.5 (IQR 5; 20) cigarettes per day for 39.5 (IQR 27.5; 41.3) years. Former smokers smoked a median of 20 (IQR 20; 20) cigarettes for 20 (IQR 15; 40) years before they quit. Current smokers smoked from 4.5 to 40 pack-years, median 25, whereas former smokers smoked from 1 to 90 pack-years, median 22.5. No patients reported waterpipe or electronic cigarette use in the group. Not only was cigarette smoking prevalence low, but other environmental risk factors, such as exposure to SHS or use of organic fuel for heating, were reported by only a quarter of the included patients. Finally, we found that only 6% exercised regularly.

Clinical and symptom burden was high in the cohort, including respiratory symptoms demonstrating high CAT and mMRC scores, high respiratory rate and low SpO_2_ at admission (Table 1). Spirometry found a reduction in volumes in the majority of included patients, with the FVC median corresponding to mild impairment (−2.17 z-score). An obstructive defect was found in a very small fraction of patients, and the FEV1/FVC was quite high in the group (Table 1). The ILD-IPF group differed from the ILD-SD group in demographic (fewer women and generally older population), lifestyle and even symptom attributes. The former had greater CAT and mMRC scores, as well as SpO_2_ and even respiratory rate. Most spirometric parameters had a tendency for greater reduction in them, but these differences failed to reach statistical significance. VC reduction was most notable amongst the measured parameters. 

The overall maneuver quality in the group was high, despite challenges in performance. Most patients experienced some cough and severe dyspnea, increasing the need for breaks to rest between maneuvers. They exhaled from two to six times for a VC maneuver (median 3, IQR 3; 3) (Table 2), whereas ΔVC ranged from 0 to 0.24 L. There were more attempts at an FVC maneuver overall, two to 11, median 3 (IQR 3; 4). ΔVC (median 0.05 L) was comparable to ΔFVC (median 0.07 L) and even ΔFEV1 (0.05 L). BEV fell within the acceptable range in most cases. The expiratory plateau volume had a median of 0.04 L, which was above the recommended 25 mL for an acceptable curve; however, most tests (96%) were graded either A or B quality by ΔFVC and even more tests (98%) were graded A or B quality by ΔFEV1. When we compared maneuver quality by the indicators listed in Table 2 between patients with no defect, a suggestion of a restrictive pattern and an obstructive defect, we found no differences in the number of attempts. However, repeatability criteria compliance was better in the suggestion of a restriction patients when ΔVC was considered, and no differences in ΔFVC or ΔFEV1 were found (Table 2). Finally, the median Δ(VC-FVC) was 0.06, but up to one-third of the group had Δ(VC-FVC) exceeding 100 mL, much greater than in the obstructive group. Although the differences in the number of patients with Δ(VC-FVC) exceeding 100 mL were not significant, the spirometries suggestive of a restriction tended to have the highest quality for this quality criterion. 

We further tested the predictors of VC and FVC maneuver quality listed in Table 2, first in the univariate models. These predictors of interest included age, sex, BMI, cigarette smoking, BMI, CAT and mMRC scores, SaO_2_, HR, ILD-IPF vs. ILD-SD, and respiratory rate at admission. We found that, with regard to VC maneuver, only the CAT score and HR were associated with the number of attempts, but no associations were found for ΔVC. However, the range of predictors for FVC maneuver quality was much wider. Thus, mMRC and CAT scores were associated with the number of attempts, whereas age and sex showed significant associations with ΔFVC. mMRC was the only predictor for ΔFEV1. Expiratory plateau volume was associated only with the CAT score and HR. For Δ(VC-FVC), no associations with any tested variables were identified. Finally, the range of variables was widest for BEV and included the respiratory rate, SaO_2_, mMRC and CAT scores, BMI sex and even smoking. In the adjusted models, none of the selected predictors showed significant associations with the number of both VC and FVC maneuver attempts, or with ΔVC, expiratory plateau volume and BEV. However, we found that ΔFVC was positively associated with male sex (adjusted for age β-coefficient 0.03, 95% CI 0.01; 0.05), but the effect was weak; ΔFEV1 had a significant, but very weak association with the mMRC score (crude β 0.01; 95% CI 0.00; 0.02).

## 4. Discussion

This cross-sectional study of spirometry quality in a group of prospectively enrolled ILD patients, admitted to a tertiary-level facility for hospitalization, found that, overall, the quality of spirometry was good. These patients usually suffer from persistent dyspnea and other respiratory symptoms, which hamper successful lung function testing completion. Nevertheless, this study showed that A and B grades can be achieved in the majority of such patients. We failed to identify significant predictors of the tests’ high quality with a notable effect, making quality criteria attainment a matter of individual variability and compliance. 

Spirometry quality is a cornerstone for obstructive ventilation defect verification, and spirometry should be first used to identify chronic obstructive pulmonary disease (COPD) [19]. The greatest patient load rests with the primary care facilities; therefore, studies to report spirometry quality concentrate mainly in these [20,21]. Spirometry quality from an array of primary centers differs dramatically, and there often exists some disagreement between general practitioners and pulmonologists at the interpretation stage [21]. Studies from in-patient centers can also be found in the literature, and they usually report a moderate amount of acceptable spirometries [22]. Recently, an outbreak in artificial intelligence is believed to have improved the interpretation, and various deep learning models are now being tested around the world [23,24,25]. However, the technical aspect of spirometry testing is still dependent on humans, and proper instructions and encouragement to exhale forcefully still rest with an instructor. Artificial intelligence tools may help identify spirometric patterns, but may not be of much use in the testing room to perform maneuvers, although deep learning is also reported to improve spirometry quality with more curves graded A or B [26].

Patients with chronic disease with a heavy burden of symptoms, such as ILD, or the elderly, may be a challenging group in which to attain high quality, because they reach fatigue earlier, may have a lower threshold for hypocapnia with dizziness from a deep breath, and often cognitive reduction has been shown to predict poorer spirometry quality in the elderly [27]. Another study also showed higher odds of poor-quality spirometry in subjects with worse cognitive function [28]. Furthermore, muscle weakness, important for high-quality spirometry, was possibly identified as a peril in achieving a sufficient number of acceptable curves in patients with many chronic and debilitating conditions, including systemic sclerosis [29]. This may often be the case for patients with ILD. Studies specifically planned to assess spirometry quality in ILD patients are not abundant; therefore, our presentation can fill the gap in understanding how such patients accomplish spirometry given that they suffer from advanced dyspnea, cough and fatigue.

Overall, we demonstrated that spirometry quality in ILD can also be high in the majority of patients, and an A or B grade for ΔFVC could be achieved in 96% of patients. Nevertheless, up to one-third of ILD patients in our study showed increased Δ(VC-FVC), indicative of some worse reproducibility, and this criterion should also be taken into account when grading spirometry quality during test interpretation. There were more patients attaining a high quality in our ILD group compared to a population-based study in Kazakhstan in 2025, in which only 78% of patients were graded A or B for ΔFVC. We believe that proper quality, even in patients with a high respiratory burden, such as IPF patients, can be better attained in a tertiary-level facility with more time available, higher standards for regular personnel training, stricter criteria for control and maintenance and greater instructor experience. These two studies can be compared directly, because they are from one source population. We conclude that in respiratory-compromised patients, spirometry quality may still be very high. Nevertheless, criteria such as respiratory plateau volume and BEV need more attention. 

Compromised cognitive function [28] was not the only reported predictor for poorer spirometry quality, but also male sex and even lower income [30]. In our group of ILD patients, we also found that male sex was significantly associated with greater ΔFVC, reducing spirometry quality, but the effect was very weak overall. Further studies should take into account more individual factors that could affect spirometry quality, because their identification may help find patients in need of additional instruction and a more personalized approach in attaining sufficient quality. Large cohort studies, such as in large occupational groups [31,32], report spirometry in well-trained groups of thousands of people, and these can serve as a better venue to test individual spirometry quality predictors; however, such studies face the perils of massive budgets and extending over time. In specific groups of patients, such as those with ILD, in whom successful high-quality spirometry completion is challenging due to cough, dyspnea and fatigue, individual quality predictors are harder to identify. 

Our study has a number of limitations. First, we did not consider bronchodilation and subsequent quality testing in dilated bronchi. Secondly, we did not supplement spirometry with subsequent body plethysmography, given that most patients had a pattern suggestive of restriction. Therefore, we could only confirm this ventilation defect. Thirdly, the sample was relatively small compared to some other studies elsewhere, and that could explain the non-significant associations of the selected predictors with the quality criteria, because they just did not reach sufficient power. Fourthly, we admit that the use of a CAT questionnaire in a cohort of ILD patients may not have captured the entire spectrum of respiratory symptoms, because the tool had been designed and validated in COPD patients. However, given that the tool persisted in the questionnaires used in our preceding studies and that its selected items performed well in quantifying the severity of selected symptoms, such as cough, phlegm production and even dyspnea, we retained the tool in this study. Finally, the cross-sectional design of this study could only help test our hypothesis, not confirm true associations. 

## 5. Conclusions

In this cross-sectional study in a tertiary-level facility with consecutive ILD patient recruitment, we found that most patients had a pattern suggestive of restriction, as expected. We also conclude that moderate-to-high spirometry quality in ILD patients could be achieved in the majority of patients, even despite a high respiratory symptom burden. Further studies in larger samples are needed to identify predictors of poor spirometry quality to help personalize instruction and compliance.

## Figures and Tables

**Table 1:** The overall summary of participants, including demographic, lifestyle, clinical and spirometric variables.

Variable	Overall	ILD-IPF	ILD-SD
N (%)	125 (100)	40 (32)	85 (68)
Females, N (%) *	91 (73)	17 (43)	74 (87)
Age, years *	60 (52; 67)	67 (61; 70)	58 (48.5; 64)
BMI, kg/m^2^	25.1 (22.8; 28.8)	25.2 (22.6; 30.7)	25.1 (22.8; 27.4)
Education, N (%)			
Below high school	1 (1)	0 (0)	1 (1)
High school	30 (24)	12 (30)	18 (21)
College	36 (28)	15 (38)	21 (25)
University	57 (46)	13 (32)	44 (52)
Academic degree	1 (1)	0 (0)	1 (1)
Cigarette smoking, N (%) *			
Never	90 (72)	17 (42)	73 (86)
Former	29 (23)	19 (48)	10 (12)
Current	6 (5)	4 (10)	2 (2)
Exposure to SHS, N (%)	31 (25)	12 (30)	19 (22)
Use of organic fuel for heating, N (%) *	30 (24)	14 (35)	16 (19)
Use of organic fuel for cooking, N (%)	5 (4)	0 (0)	5 (6)
Regular exercise, N (%)	8 (6)	1 (2)	7 (8)
CAT score (0–40) *	12 (9; 18)	16.5 (12; 22.5)	12 (8; 17)
mMRC score (0–4) *	2 (1; 3)	2 (1; 3)	1 (1; 2)
SpO_2_ at admission, % *	94 (90; 95)	91 (88; 94)	94 (92; 95)
Respiratory rate at rest, per minute *	20 (19; 21)	20.5 (20; 22)	19 (19; 20)
Heart rate at rest, per minute	80 (78; 89)	82 (76; 91.5)	80 (78; 88.5)
VC, % predicted	59 (47.5; 74)	54.5 (47; 69)	61 (47.5; 74.5)
VC, z-score	−3.26 (−4.04; −2.08)	−3.44 (−4.04; −2.26)	−3.02 (−4.07; −2.05)
FVC, % predicted	68 (52.5; 80.5)	65 (53.3; 79)	70 (50.5; 81)
FVC, z-score	−2.17 (−3.28; −1.4)	−2.38 (−3.19; −1.34)	−2.06 (−3.4; −1.4)
FEV1, % predicted	70 (55.5; 81)	68.5 (56.3; 80.3)	71 (52.5; 81)
FEV1, z-score	−2.01 (−3.06; −1.27)	−2.07 (−2.76; −1.27)	−1.99 (−3.24; −1.31)
FEV1/FVC, actual	82.7 (78.3; 87)	82.6 (79; 88.5)	83 (78; 87)
FEV1/FVC, z-score	0.36 (−0.36; 0.90)	0.58 (−0.4; 1.22)	0.25 (−0.35; 0.84)

Note: ILD-IPF—interstitial lung disease as idiopathic pulmonary fibrosis; ILD-SD—interstitial lung disease with systemic disease; BMI—body mass index; SHS—secondhand smoke; CAT—COPD Assessment Test; mMRC—modified Medical Research Council; VC—vital capacity; FVC—forced vital capacity; FEV1—forced expiratory volume in one second; *—*p* < 0.05 in χ^2^-test for binary variables either from 2 × 2 or 2 × 3 contingency tables or Mann–Whitney U-test for continuous variables, accordingly. Data are shown as medians with the corresponding interquartile range.

**Table 2:** Summary of spirometry quality overall and stratified by spirometric impairment.

	Overall	Normal Spirometry	Suggestive of Restrictive Pattern	Obstructive Pattern
N patients, (%)	125 (100)	40 (32)	77 (62)	8 (6)
VC maneuvers				
N attempts	3 (3; 3)	3 (3; 4)	3 (3; 3)	3 (3; 3.8)
ΔVC, median *	0.05 (0.03; 0.09)	0.07 (0.04; 0.12)	0.05 (0.02; 0.07)	0 (0; 0.07)
FVC maneuvers				
N attempts	3 (3; 4)	3 (3; 5)	4 (3; 4)	3 (3; 3.8)
ΔFVC, median	0.07 (0.03; 0.12)	0.07 (0.03; 0.13)	0.06 (0.02; 0.12)	0.06 (0.03; 0.11)
ΔFEV1, median	0.05 (0.02; 0.09)	0.06 (0.02; 0.10)	0.04 (0.02; 0.09)	0.03 (0.01; 0.06)
BEV *	0.07 (0.05; 0.09)	0.09 (0.07; 0.13)	0.06 (0.04; 0.07)	0.05 (0.03; 0.10)
V plateau, L *	0.04 (0.02; 0.06)	0.03 (0.02; 0.05)	0.04 (0.02; 0.06)	0.09 (0.05; 0.16)
N tests graded A (ΔFVC)	112 (90)	37 (93)	71 (92)	7 (88)
N tests graded B (ΔFVC)	7 (6)	2 (5)	3 (4)	1 (12)
N tests graded A (ΔFEV1)	116 (93)	37 (93)	72 (94)	7 (88)
N tests graded B (ΔFEV1)	6 (5)	2 (5)	3 (4)	1 (12)
VC to FVC comparison				
Δ(VC-FVC), median	0.06 (0.03; 0.15)	0.07 (0.03; 0.15)	0.06 (0.03; 0.11)	0.19 (0.03; 0.50)
N patients with Δ(VC-FVC) > 100 mL	40 (32)	15 (38)	20 (26)	5 (63)

Note: BEV—back-extrapolated volume; VC—vital capacity; FVC—forced vital capacity; FEV1—forced expiratory volume in one second; data are presented as medians with the corresponding interquartile ranges; between-group comparisons are from either 2 × 3 tables χ^2^-test or Kruskall–Wallis test; *—*p* < 0.05

## Data Availability

The original contributions presented in this study are included in the article. Further inquiries can be directed to the corresponding author.

## Abbreviations

The following abbreviations are used in this manuscript:

BEV	Back extrapolation volume
BMI	Body mass index
CAT	COPD Assessment Test
CI	Confidence interval
COPD	Chronic obstructive pulmonary disease
CT	Computer tomography
FEV1	Forced expiratory volume in one second
FVC	Forced vital capacity
HR	Heart rate
ILD	Interstitial lung disease
IPF	Idiopathic pulmonary fibrosis
IQR	Interquartile range
LLN	Lower limit of normality
mMRC	Modified Medical Research Council
SD	Systemic disease
SHS	Secondhand smoke
VC	Vital capacity
